# Carriage of mutations R462Q (rs 486907) and D541E (rs 627928) of the *RNASEL* gene and risk factors in patients with prostate cancer in Burkina Faso

**DOI:** 10.1186/s12920-022-01279-9

**Published:** 2022-06-02

**Authors:** Essonan Kadanga, Abdou Azaque Zouré, Théodora M. Zohoncon, Lassina Traoré, Bienvenu Désiré Ky, Albert Théophane Yonli, Djé Djénèba Aïda Traoré, Bapio Valery Jean Télesphore Elvira Bazié, Herman Karim Sombié, Pegdwendé Abel Sorgho, Sessi Frida Appoline Tovo, Kalifou Traoré, Teega-Wendé Clarisse Ouedraogo, Florencia W. Djigma, Jacques Simpore

**Affiliations:** 1 Laboratory of Molecular and Genetic Biology (LABIOGENE), Joseph KI-ZERBO University, 03 BP 7021 Ouagadougou 03, Burkina Faso; 2 Pietro Annigoni Biomolecular Research Center (CERBA), 01 BP 364 Ouagadougou 01, Burkina Faso; 3 Biomedical Research Laboratory (LaReBio), Biomedical and Public Health Department, Institute for Research in Health Sciences (IRSS/CNRST), 03 BP 7192 Ouaga 03, Burkina Faso; 4 Faculty of Medicine, Saint Thomas Aquinas University (USTA), 06 BP 10212 Ouagadougou 06, Burkina Faso; 5 Urology Department, CHU Yalgado Ouedraogo, UFR SDS, Joseph KI ZERBO University, 03 BP 7021 Ouagadougou 03, Ouagadougou, Burkina Faso; 6 Institute for Research in Applied Sciences (IRSAT/CNRST), 03 BP 7192 Ouaga03, Burkina Faso

**Keywords:** *RNASEL*, R462Q, D541E, Prostate cancer, Burkina Faso

## Abstract

**Background:**

Prostate cancer (Pca) is a public health problem that affects men, usually of middle age or older. It is the second most common cancer diagnosed in men and the fifth leading cause of death. The *RNASEL* gene located in 1q25 and identified as a susceptibility gene to hereditary prostate cancer, has never been studied in relation to prostate cancer in Burkina Faso. The aim of this study was to analyze the carriage of *RNASEL* R462Q and D541E mutations and risks factors in patients with prostate cancer in the Burkina Faso.

**Methods:**

This case–control study included of 38 histologically diagnosed prostate cancer cases and 53 controls (cases without prostate abnormalities). Real-time PCR genotyping of R462Q and D541E variants using the TaqMan® allelic discrimination technique was used. Correlations between different genotypes and combined genotypes were investigated.

**Results:**

The R462Q variant was present in 5.3% of cases and 7.5% of controls. The D541E variant was present in 50.0% of cases and 35% of controls. There is no association between R462Q variants (OR = 0.60; 95%IC, 0.10–3.51; *p* = *0.686*) and D541E variants (OR = 2.46; 95%IC, 0.78–7.80; p = *0.121*) and genotypes combined with prostate cancer. However, there is a statistically significant difference in the distribution of cases according to the PSA rate at diagnosis (p ˂ 0.001). For the Gleason score distribution, only 13.2% of cases have a Gleason score greater than 7. There is a statistically significant difference in the Gleason score distribution of cases (*p ˂ 0.001*).

**Conclusions:**

These variants, considered in isolation or in combination, are not associated with the risk of prostate cancer.

## Background

Prostate cancer (Pca) is the second most frequently diagnosed malignant tumour in humans in the world. It is the fifth leading cause of cancer death in humans, with an estimated 1.4 million new cases and 375,304 deaths in 2020 in the world. In Burkina Faso, in the same year, the number of new cases of prostate cancer was 997 out of 4,305 new cases of cancer, with 608 deaths caused. It is the first cancer in terms of incidence in men (and the fifth cancer in both sexes), followed by liver cancer [1]. The etiology of prostate cancer has been the subject of numerous studies but remains largely unknown. The risk factors that remain well established are advanced age, ethnicity, family history [2–4]. Indeed, the incidence of prostate cancer is estimated to be 1 in 350 for men under 50 years [5]; 1 in 52 for 50- to 59-year-olds; then 60% in men over 65 years. Almost 30% of men over 50 years who died from causes other than prostate cancer have been shown to have histological evidence of prostate cancer at the time of the autopsy [6]. Populations of African descent, such as African Americans, Caribbean, and blacks in Europe had the highest incidences, early disease and more aggressive form compared to other racial and ethnic groups [7]. Men of African descent are estimated to have a relative risk of 9.7 versus 3.9 in Caucasians and 1.6 in Asians when two or more first-degree relatives have prostate cancer [8]. Regarding family history, more than 20% of patients with prostate cancer report a family history. This can be explained on the one hand by the common sharing of genes; but also on the other hand by a similar pattern [9] of exposure to certain environmental carcinogens and to common lifestyles [10]. The relative risk of prostate cancer for men with a first-degree relative with prostate cancer is estimated to be about 2.5. This risk increases to 5.3 when three or more first-degree relatives are affected. Serum prostate antigen assay and rectal touch are currently the primary screening methods for prostate cancer [11]. However, with the ultimate goal of developing new, more accurate and beneficial biomarkers in the detection, prevention and treatment of this disease, several studies have been conducted to elucidate the molecular mechanisms involved in the genesis and progression of prostate cancer [12]. The high incidence of prostate cancer in African men suggests a genetic predisposition. Initial quantitative genetic analyses of homo and dizygous twins estimated that germ mutations contributed to prostate cancer risk at approximately 40–58% [13–15]. Linkage analysis and positional cloning were used to successfully map inherited chromosomal regions containing prostate cancer susceptibility genes. The HPC1 (Hereditary Prostate Cancer 1) locus, located on chromosome region 1q24-25, was the first of these loci to be identified in 1996 [16, 17]. Since then, several other loci of predisposition to hereditary forms of prostate cancer have been identified: HPCX (Xq27-28), HPC20 (20q13), HPC2 (17p11), PG1 (prostate cancer susceptibility gene 1) (8p22-23), CAPB (1p36) [18]. Three genes for hereditary prostate cancer susceptibility have been identified in three of these loci. This is the *RNASEL*(2'-5' oligoadenylate synthetase-dependent ribonuclease) gene (HPC1); of ELAC2 (ElaC Ribonuclease Z 2) (HPC2) which encodes a metallo-dependent hydrolase potentially involved in the repair of the inter-strand cross-linking of DNA and the editing of mRNA and finally the MSR1(Macrophage Scavenger Receptor1) (PG1) gene which encodes subunits a macrophage scavenger receptor which is capable of binding to a variety of ligands. [19–23]. Mutations in these different genes have low or moderate penetrance. They influence the way the prostate works and are responsible for about 30% of prostate cancer [24]. Other high penetration mutations have been identified in the genes regulating: the critical stages of the development process, namely the G84E mutation of the *HOXB13* gene [25, 26]; the Q356R, 185delAG, 5382insC and 6174delT mutations in the *BRCA2* gene [27]. Studies of these different regions related to prostate cancer in different populations have provided inconsistent results. These observations show the genetic complexity and heterogeneity (environmental and genetic factors) of prostate cancer predisposition. The *RNASEL* gene, located at 1q24-25, with a size of about 15 kilos pair of bases, and comprising 8 exons; code for ribonuclease 2'-5'-oligoadenaylate (2-5A) -dependent. *RNASEL* regulates cell proliferation and apoptosis through the interferon-induced 2’-5’A pathway through its antiviral and antiproliferative activity [28]. There are many nucleotide variants identified in the *RNASEL* gene. Seven of them cause changes in the protein sequence. Six variants cause false sense alterations and a rare variant creates a nonsense mutation [29]. The most commonly studied synonymous variants in association with prostate cancer in different types of populations are R462Q and D541E. The different expression studies did not prove that the two polymorphisms of RNASEL can influence the expression of the gene; but the functional studies were able to show that the R462Q reduces the ability of the cell to cause apoptosis in response to 2’-5’A activation and also has three times less enzymatic activity than normal, while D541E does not affect the function of the Rnase L protein [18, 19]. The results of these studies remain contradictory. The AA genotype in R462Q has been associated with both an increased risk of prostate cancer in the United States and in some Caucasian population groups [30, 31] and a decreased risk in Caucasian and Japanese sample groups. Previous studies on the *RNASEL* variant D541E indicated that the GG and TT genotypes were associated with an increased risk of prostate cancer in some Japanese [32] and European-American [33] populations, respectively. On the other hand, a negative association of the TT genotype with prostate cancer in Swedish Caucasian samples was reported by Wiklund et al*.* in 2004 [34]. In summary, several studies provide strong support, both functional and epidemiological, that *RNASEL* plays a role in prostate cancer, but other studies have suggested a lack of role based on the ethno-geographic origins of study populations. In West Africa, several studies of prostate cancer in different populations have focused on the epidemic and morphological aspects of prostate cancer [35–37]. Very few studies have examined the genetic background of African populations and its contribution to prostate cancer susceptibility. This limits the use of genetic data at all levels of prostate cancer management such as screening, diagnosis, treatment and follow-up in the African context. The study described here was undertaken to determine the involvement of R462Q and D541E variants of the *RNASEL* gene in prostate cancer in the Burkinabe population. This could provide additional information that could potentially be exploited to improve early detection and diagnosis of high-risk individuals for early therapeutic intervention or ease of management.

## Materials and methods

### Design of study

The study was conducted between October 2019 and April 2021. The study population (Burkinabe) consisted of 38 patients, histologically diagnosed with prostate cancer (cases) and 53 males at least 45 years of age with either a total PSA levels less than 4 ng/ml or normal PSA derivatives (free PSA, free / total ratio, velocity and density of PSA) or a negative prostate biopsy (controls). They are all monitored at the Saint Camille hospital in Ouagadougou (HOSCO) or at the NINA clinic in Ouagadougou. Biomolecular analyzes were carried out at the Molecular and Genetic Biology Laboratory (LABIOGENE) and at the Pietro Annigoni Biomolecular Research Center (CERBA).

### Sample collection

After obtaining consent from patients and controls, a questionnaire was administered to collect sociodemographic, anthropometric, and clinical data from participants. Venous blood from consenting participants was collected on Ethylene- Diamine-Tetra-Acetic (EDTA) filled tubes. After centrifugation, at 3,500 revolutions per minute for 15 min, the plasma and pellets were separated and stored at − 20 °C.

### PSA assay

PSA levels were assayed at the HOSCO laboratory on the Cobas 6000 automated system using the "Elecsys Total PSA" reagent. This test is an “ECLIA” electro chemiluminescence immunoassay. It is based on the “sandwich” method.

### DNA extraction and genotyping

The DNA was isolated from the total blood of the participants by the ‘salting out’ technique as described by Miller et al*.* (1988). TaqMan® allelic discrimination was used to genotype nucleotide variants R462Q (rs486907) and D541E (rs627928) of the *RNASEL* gene. The primers and probes for R462Q were as follows [38]: forward primer 5’-GGAAGATGTGGAAAATGAGGAAGA-3’, reverse primer 5’-TGCA GATCCTGGTGGGTGTA-3’, and probes 5’-***VIC-***CAGGACATTTCGGG CAA-***MGB*** and 5’-***FAM-***CAGGACATTTTGGGCAA-***MGB***. Primers and probes for D541E were as follows: forward primer 5'-TCTATGTGGTAAAGAAGGGAAGCA-3’, reverse primer 5’-TTGAAC CACCTCTTCATTACTTTGAG-3’ and probes 5’-***VIC***-TTTCAGATCCT CAAAT***-MGB*** and 5’-***FAM***-TTTCAGCTCCTCAAAT***-MGB***.

**Fig. 1:**
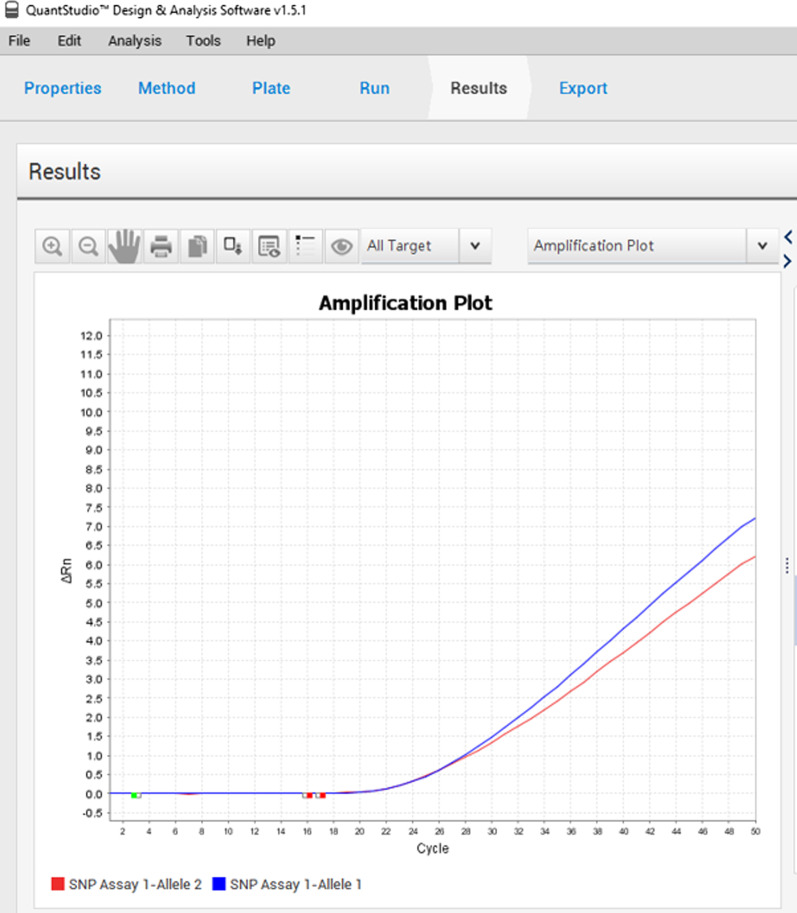
Curve of wild (blue) and mutated (red) genotypes

The target sequences were amplified by Real-time PCR in a 25 µL reaction mixture consisting of 5 µL of DNA, 1 µL of each primer at 200 nmol/L and 0.2 µL of each probe at 900 nmol/L, 8 µL of TaqMan® Universal PCR Master Mix II 2X (Applied Biosystems), and the remainder is completed with sterile water. PCR were run on a 95 °C program for 10 min followed by 50 cycles of denaturing at 95 °C for 15 s and hybridization/extension at 60 °C for 1 min on QuantStudio 5 (Applied Biosystems) detection system. TaqMan® Genotyper 1.6.0 software (Applied Biosystems) was used to determine genotypes (Fig. 1).

### Statistical analyses

Data was entered using Excel 2016 software. For each polymorphism, allelic frequencies were determined and compared between cases and controls using the Chi^2^ and Fisher exact tests. Hardy Weinberg’s equilibrium was checked for each polymorphism. In order to verify the association between each polymorphism of the *RNASEL* gene and the risk of prostate cancer, the Odds ratios (OR) and 95% confidence intervals (95% CI) were determined by considering the age at the time of the cancer diagnosis in the cases. The analyses were carried out using R 4.1.1 software. Analyses were considered statistically significant at *p* ≤ *0.05*.

## Results

### Socio-demographic characteristics of the study population

The characteristics of our study population are presented here in (Table 1). The mean age in years of the cases at the time of study was 69.81 ± 8.05 and 65.49 ± 8.90 that of the controls. The distribution by age at diagnosis shows that 60.5% of cases were diagnosed between 51 and 70 years old and 39.5% at over 70 years old. The average age at diagnosis is 67.13 ± 8.17 years. There is no statistically significant difference between the mean age at the diagnosis and that of the controls (p = 0.365). With regard to the family history of prostate cancer, 36.8% of cases and 32.1% of controls have a family history.

**Table 1:** Socio-demographic characteristics

Subgroup	Cases (n = 38) n (%)	Controls (n = 53) n (%)	*P*-value
Age during the study (years)			
≤ 50	0 (0)	5 (9.4)	
51–70	17 (44.7)	35 (66.0)	
> 70	21(55.3)	13 (24.5)	
Mean (SD)	69.81 (8.05)	65.49 (8.90)	**0.017**
Age at diagnostic (years)			
≤ 50	0 (0)		
51–70	23 (60.5)		
> 70	15 (39.5)		
Mean (SD)	67.13 (8.17)		
Family history			
Yes	14 (36.8)	17 (32.1)	
No	18 (47.4)	25 (47.2)	
Unknown	6 (15.8)	11 (20.8)	

statistically significant, *p* ≤ 0.05 is shown in bold

*SD* standard deviation

### Biological characteristics of the study population

The distribution according to the PSA levels at diagnosis shows that the majority of cases, i.e. 81.6%, have a PSA level at diagnosis greater than 20 ng/ml. There is a statistically significant difference in the distribution of cases according to the PSA rate at diagnosis (p ˂ 0.001). For the Gleason score distribution, only 13.2% of cases have a Gleason score greater than 7. There is a statistically significant difference in the Gleason score distribution of cases (*p ˂ 0.001*) (Table 2).

**Table 2:** Biological characteristics

Subgroup	Cases (n = 38) n (%)	Controls (n = 53) n (%)	*P*-value
PSA during this study (ng/ml)			
≤ 4.0		37 (70)	
4.1–10.0		11 (21)	
10.1–20.0		3 (6)	
> 20		2 (3)	
Mean (SD)		4.16 (4.70)	
PSA at diagnosis (ng/ml)			
≤ 4.0	0 (0)		
4.1–10.0	3 (7.9)		
10.1–20.0	4 (10.5)		< **0.001**
> 20	31 (81.6)		
Mean (SD)	627.85 (1153.42)		
Gleason score			
< 7	10 (26.3)		
7	23 (60.5)		< **0.001**
> 7	5 (13.2)		

statistically significant, *p* ≤ 0.05 is shown in bold

*PSA* prostate specific antigen, *SD* standard deviation

### Prostate cancer and lifestyle

No association between risk of prostate cancer and lifestyle such as physical inactivity (*p* = *0.31*), alcohol intake (*p* = *0.80*), smoking (*p* = *0.62*), and the consumption of fatty meat (*p* = 0.67) (Table 3).

**Table 3:** ORs for lifestyle and prostate cancer risk

	Cases (%)	Controls (%)	OR	IC 95%	*P*-value
Physical activity					
Yes	22 (73,3)	43 (82,7)	1	Reference	
No	8 (26,7)	9 (17,3)	1,74	0,59 – 5,13	0,314
Alcohol					
No	13 (43,3)	24 (46,2)	1	Reference	
Yes	17 (56,7)	28 (53,8)	1,12	0,45–2,77	0,805
Smoking					
No	25 (83,3)	41 (78,8)	1	Reference	
Yes	5 (16,7)	11 (21,2)	0,75	0,23–2,40	0,621
Fatty meat					
No	12 (33,3)	20 (37,7)	1	Reference	
Yes	24 (66,7)	33 (62,3)	1,21	0,50–2,95	0,671

*OR* Odd Ratio, *CI* confidence interval

### Allelic frequencies

For the R462Q mutation, the [G] allele was the most frequent allele in both the case population 0.868 and the control population 0.802. There was no statistically significant difference between allele frequencies in cases and controls (*p* = *0.959*). Conversely, the [G] allele for the D541E variant was more prevalent among cases 0.671 and controls 0.538. No statistically significant difference was also observed between allele frequencies in cases and controls (*p* = *0.881*) (Table 4). The two polymorphisms studied were in Hardy–Weinberg equilibrium in the control population (*p* = *0.193* and *p* = *0.203*).

**Table 4:** Alleles frequencies

SNP	Allèle	Cases (n = 38)	Controls (n = 53)	*P*-value
*RNASEL* R462Q	G	0.868	0.802	0.959
	A	0.132	0.198	
*RNASEL* D541E	T	0.329	0.462	0.881
	G	0.671	0.538	

### Associations of SNPs RNASEL R462Q and D541E with prostate cancer risk

No statistically significant association between the R462Q mutation and the risk of prostate cancer was found in our study population (OR, 0.60; 95% CI, 0.10–3.51; *p* = *0.686*) (Table 5).

**Table 5:** ORs for *RNASEL* 462 SNP and prostate cancer risk

Genotypes	Cases (n = 38) n (%)	Controls (n = 5 3) n (%)	OR (95% CI)	*P*-value
GG	30 (78.9)	36 (67.9)	1.0 (Reference)	
AG	6 (15.8)	13 (24.5)	0.55 (0.19–1.63)	0.281
AA	2 (5.3)	4 (7.5)	0.60 (0.10–3.51)	0.686
AA vs AG/GG (Rec A)			0.68 (0.12, 3.92)	1.000
AA/AG vs GG (Dom A)			0.56 (0.21, 1.49)	0.245

*Rec* recessive, *Dom* dominant, *OR* odds ratios, *CI* confidence interval

The result found no statistically significant association between the D541E mutation and the risk of prostate cancer in our study population (OR, 2.46; 95% CI 0.78–7.80; *p* = *0.121*) (Table 6).

**Table 6:** ORs for *RNASEL* 541 SNP and prostate cancer risk

Genotypes	Cases (n = 38) n (%)	Controls (n = 53) n (%)	OR (95% CI)	*P*-value
TT	6 (15.8)	14 (26.4)	1.0 (Reference)	
TG	13 (34.2)	21(39.6)	1.44 (0.44–4.70)	0.541
GG	19 (50.0)	18 (34.0)	2.46 (0.78–7.80)	0.121
GG vs GT/TT (Rec G)			1.94 (0.83, 4.56)	0.125
GG/GT vs TT (Dom G)			1.91 (0.66, 5.55)	0.227

*Rec* recessive, *Dom* dominant, *OR* odds ratios, *CI* confidence interval

### Combined genotypes of RNASEL R462Q and D541E linked to prostate cancer

This study found no statistically significant association between the risk of prostate cancer and the different combinations of genotypes of the mutations of the R462Q and D541E polymorphisms (Table 7).

**Table 7:** ORs for *RNASEL* 462/541 combined genotypes and prostate cancer risk

Combined genotypes 462/541	Cases (n = 38)	Controls (n = 51)	OR (95% CI)	*P*-value
GG/TT	6	13	1.0 (Reference)	
GG/GT	12	14	1.85 (0.54–6.40)	0.497
GG/GG	12	9	2.89 (0.79–10.57)	0.192
AG/GG	5	7	1.52 (0.34–6.94)	0.852
AG/GT	1	6	0.36 (0.00–4.33)	0.628
AA/GG	2	2	2.16 (0.12–35.61)	0.589

### Associations of SNPs RNASEL R462Q and D541E with Gleason score

The R462Q and D541E mutations were compared between patients according to the Gleason score (≤ 7 and ˃ 7).

**Fig. 2:**
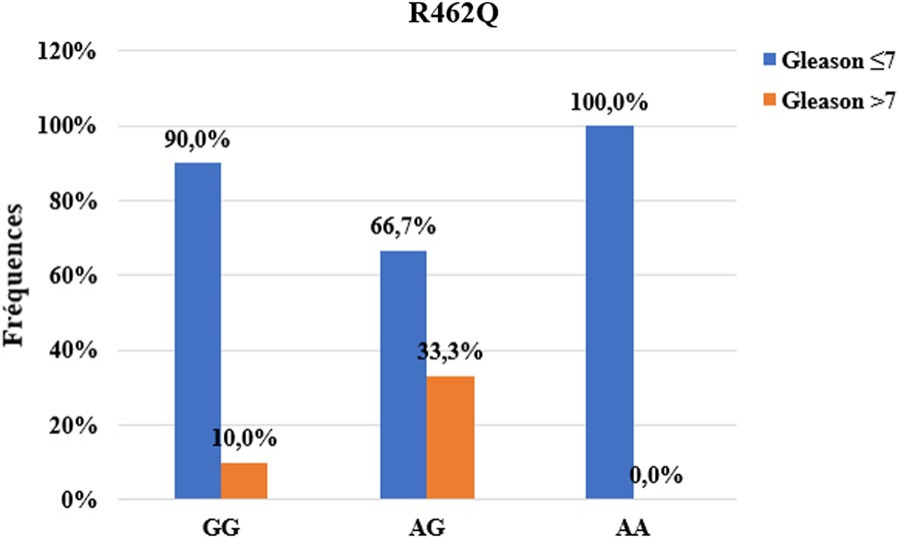
Association between Gleason score and R462Q mutation

For the R462Q mutation, 33.3% of carriers of the AG genotype and 10.0% of carriers of the GG genotype have a Gleason score greater than seven (7) while 100% of carriers of AA genotypes have a score of seven at more (Fig. 2). We found a statistically significant association between the R462Q mutation and the Gleason score (*p ˂ 0.001*).

**Fig. 3:**
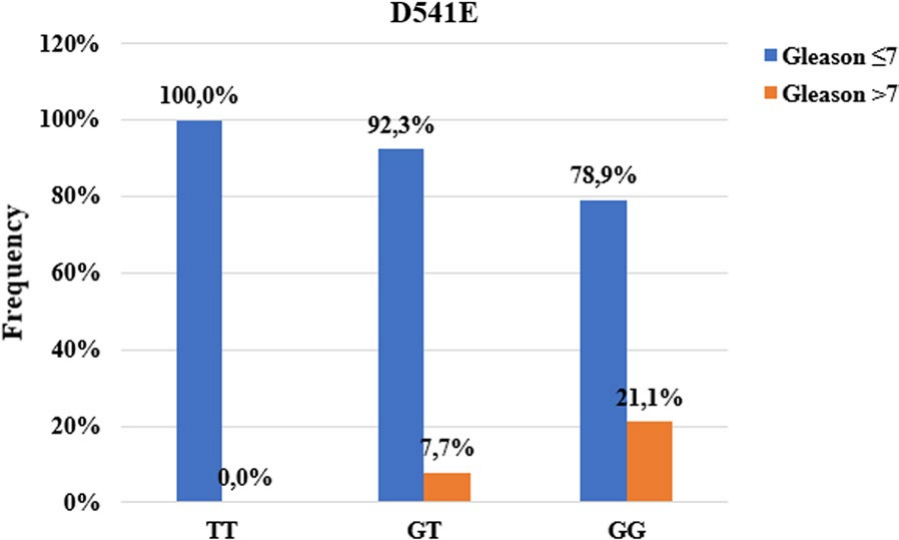
Association between Gleason score and D541E mutation

For the D541E mutation, 21.1% of carriers of the GG genotype and 7.7% of carriers of the GT genotype had a Gleason score greater than 7 while 100% of carriers of TT genotypes had a score of 7 at more (Fig. 3). We found a statistically significant association between the D541E mutation and the Gleason score (*p ˂ 0.001*).

### Associations of SNPs RNASEL R462Q and D541E with PSA at diagnosis

**Fig. 4:**
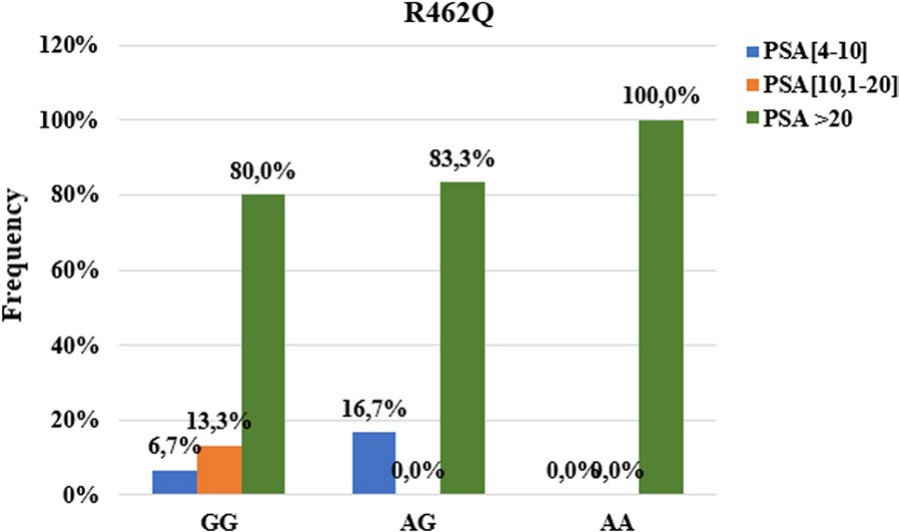
Association between PSA level at diagnostic and R462Q mutation

The PSA levels at diagnosis according to the different genotypes of the R462Q mutation indicate that 100% of carriers of the AA genotypes have PSA greater than 20 ng / ml. Only carriers of the GG genotype (13.3%) present PSA levels between 10.1 and 20 ng / ml (Fig. 4). No association was found between this mutation and the PSA level at diagnosis greater than 20 ng / ml (*p* = *0.773*).

**Fig. 5:**
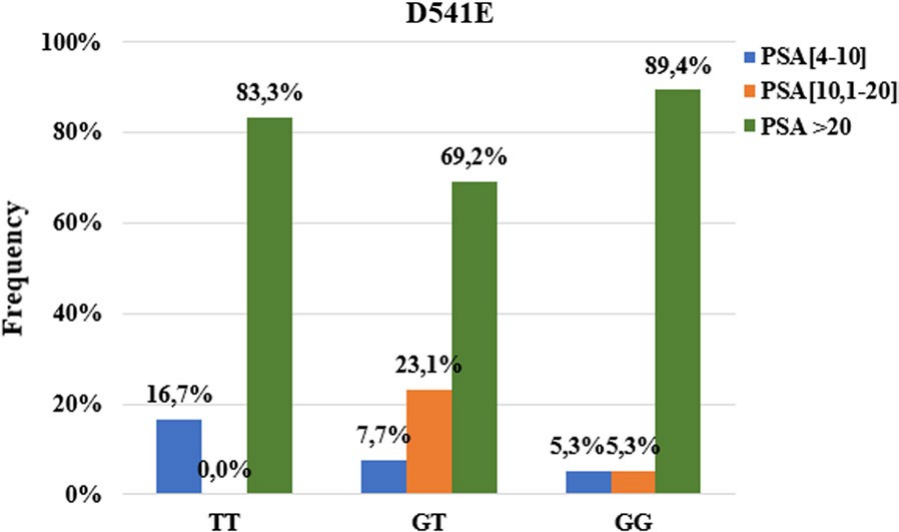
Association between PSA level at diagnostic and D541E mutation

For the D541E mutation, 89.4% of the GG genotype have a PSA level greater than 20 ng / ml. 23.1% of carriers of the heterozygous GT genotype and 5.3% of carriers of the mutated GG genotype had a PSA level at diagnosis between 10.1 and 20 ng / ml (Fig. 5). No association was found between this mutation and PSA levels at diagnosis greater than 20 ng / ml *(p* = *0.346*).

## Discussion

Sociodemographic characteristics of the study participants show that the mean age at diagnosis of cases was high, at 67.13 ± 8.17 years. This result is not different from that of Kaboré et al*.* [39] who report an average age of 71.5 years in Burkina Faso. These results indicate that the age at diagnosis in Burkina Faso is high as observed elsewhere in West Africa [40]. But our results are contrary to those obtained in various studies reporting when black men have an age at early diagnosis [41–43]. PSA levels at diagnosis were very high in our study with a mean of 627.85 ± 1153.42 ng / ml. Our results are in agreement with those obtained by Kaboré et al*.* in Burkina Faso with an average PSA of 537 ng / ml [35]. Our results corroborate those of Niang et al*.* in Senegal and Ofoha and Magnus in Nigeria [37, 44]. Tengue et al*.* in Togo also found PSA levels at diagnosis greater than 100 ng / ml [36]. Among the cases with their Gleason score, 82.14% have a score less than or equal to 7. This shows that the majority of these cases presented with a moderately differentiated tumor at diagnosis. These different results suggests that the diagnosis of prostate cancer is made at advanced stages of the disease and, the fact that there is absence of prostate cancer screening programs in this setting. Regarding the family history of prostate cancer, of the 32 cases with a family history, 43.8% have a family history while 56.2% did not. These results could show that the majority of prostate cancer cases in our study population are not familial. But this trend could be due to the fact that the information was collected through verbal testimonies and not from medical records. Indeed, patients could confuse other prostate conditions (example benign hypertrophy) and prostate cancer.

Regarding alcohol consumption, our results are similar to those obtained by Dennis et al*.* [45] who found only a strong association between alcohol consumption and prostate cancer mortality. Our results do not support those obtained by Middleton et al*.* in 2009 [46] and Rota et al*.* in 2012 [47] in their meta-analyzes. Concerning cigarette smoking, our results are different from those obtained by Jones et al*.* in England; Cerhan et al*.* in the United States and Giovannucci et al*.* also in the United States [48–50]. All these different studies have only shown a slight increase in the risk of developing prostate cancer while a strong association was found with mortality. No association was found between physical activity and prostate cancer in our study. Our results do not corroborate those of Guéritat in France. This study demonstrated that physical exercise prevents the progression of prostate cancer either by regulating redox status and redox-dependent signaling pathways, or via the modulation of cholesterolemia or even of the expression profile of miRNAs [51]. Considering the consumption of fatty meat, our results corroborate those of Park et al*.* in their study of a population of Hawaii and Los Angeles and those of Dennis et al*.* in their meta-analysis of 4 cohort studies [45, 52].

Linkage analyzes of families at high risk for prostate cancer have provided convincing evidence that the HPC1 locus is likely to harbor a prostate cancer susceptibility gene [53]. The *RNASEL* gene has been proposed as a putative tumor suppressor gene located in this region by the positional cloning technique and by the candidate gene approach [54]. Association analyzes of the R462Q and D541E variants within the *RNASEL* gene with the Prostate cancer have achieved controversial results. Analysis of the different genotypes of the R462Q variant in our study population showed no association of this variant with prostate cancer. Our results support the conclusions of Wei et al*.*; Noonan et al*.*; and Alvarez et al*.* [21, 29, 30] as well as those of Fredrik et al*.* [34]. These studies found no association between the R462Q variant and prostate cancer. However, Casey et al*.* and Xiang et al*.* [30, 31] show that the AA genotype of the R462Q variant is significantly associated with prostate cancer. Regarding the D541E variant, our study found no association with prostate cancer. This goes hand in hand with the studies of Wei et al*.*; Ignacio et al*.*; Shook et al*.* [38, 55, 56] as well as several other authors [29, 30, 57, 58]. Contrary to our results, Noonan-Wheeler et al*.* and Wiklund et al*.* [33, 34] in a Swedish population observed an association between the GG genotype and an increased risk of prostate cancer.

Our results showed an association between the R462Q mutation and the degree of tumor differentiation (p ˃ 0.001). Indeed, carriers of heterozygous AG genotype (33.3%) and normal GG genotype (10.0%) presented undifferentiated tumors (Gleason ˃7) unlike carriers of mutated genotype. Our results are identical to those obtained by Alvarez-Cubero et al*.* in Spain [59]. On the other hand, San Francisco et al*.* found no association between the R462Q mutation and Gleason score [56]. For the D541E mutation, we also found an association with the degree of tumor differentiation. It can be seen that 21.1% of the undifferentiated tumors were carriers of the mutated GG genotype against 7.7% and 0% for carriers of the heterozygous and homozygous TT genotype respectively (p ˃ 0.001). The same result was obtained by San Francisco et al*.* in Chile [56]. In contrast, Alvarez et al*.* found no association between this mutation and the Gleason score [59]. We found no association between R462Q and D541E mutations with PSA levels at diagnosis. This shows that these two mutations in the *RNASEL* gene are not associated with the level of risk of the tumor (PSA level at diagnosis). Indeed, the PSA level at diagnosis makes it possible to measure the level of risk of tumor progression. For PSA values at diagnosis greater than 20 ng / ml, the tumor is considered to be associated with a high risk of progression [60].

The differences between our results and other studies may, on the one hand, be justified by the difference in sample sizes; the method of selection of controls and, on the other hand, by the ethno-geographic differences of the study populations. Indeed, the small sample size implies a lack of the statistical power to detect associations. Also, the genetic predisposition to prostate cancer is heterogeneous (contribution of environmental and genetic factors) in its hereditary form [61] and involves the predisposition genes in a variable way depending on ethno-geographic origins.

## Conclusion

Our study is a first to explore the links that could exist between the Arg46Gln and D541E variants of the *RNASEL* gene and prostate cancer in Burkina Faso. Genetically, the [G] allele of the R462Q variant and the [G] allele of the D541E variant were the most common in our study population. There is no difference in allele frequencies between cases and controls. These variants, taken alone or in combination, are not associated with the risk of prostate cancer in Burkina Faso population.

## Data Availability

The datasets used and/or analysed during the current study available from the corresponding author on reasonable request.

## Abbreviations

D541E: Aspartic acid 541glutamic acid
ECLIA: Electro chemiluminescence immunoassay
HPC1: Hereditary Prostate Cancer 1
MSR1: Macrophage Scavenger Receptor1 Pca: Prostate cancer
PSA: Prostate specific antigen
R462Q: Arginine 462 glutamine
RNASEL: 2'-5' Oligoadenylate synthetase-dependent ribonuclease
